# Qualitative analysis of the impact of Oral Potentially Malignant Disorders on daily life activities

**DOI:** 10.1371/journal.pone.0175531

**Published:** 2017-04-14

**Authors:** Jyothi Tadakamadla, Santhosh Kumar, Ratilal Lalloo, Newell W. Johnson

**Affiliations:** 1 Menzies Health Institute Queensland and School of Dentistry and Oral Health, Griffith University, Gold Coast, Queensland, Australia; 2 School of Dentistry, The University of Queensland, Brisbane, Queensland, Australia; 3 Menzies Health Institute Queensland, Griffith University, Queensland, Australia; Public Library of Science, FRANCE

## Abstract

**Objective:**

To evaluate the impact of Oral Potentially Malignant Disorders (OPMD) on daily life activities.

**Materials and methods:**

Patients diagnosed with Oral Leukoplakia, Oral submucous fibrosis and Oral Lichen Planus attending the Oral Medicine clinic of Panineeya Institute of Dental Sciences & Research Centre, Hyderabad, India were invited to participate. Eighteen interviews and three focus groups were conducted in a non-clinical setting. Voice recordings were transcribed and translated from Telugu to English. Data coding was performed using the NVivo software.

**Results:**

Sample size for this qualitative study comprised 32 patients. Four main themes emerged: (1) difficulties with diagnosis and knowledge about the condition, (2) physical impairment and functional limitations, (3) psychological and social wellbeing and (4) effects of treatment on daily life. In a majority of the patients, most of the interview time was spent discussing physical impairment and functional limitations. Patients also reported their mouth condition having a debilitating effect on their psychological well-being and social interactions.

**Conclusions:**

**‘**Physical impairment and functional limitations’ was the most important theme for many of the patients. However, the impacts of OPMD also extended beyond physical impairment and functional limitations to aspects of daily living, notably psychological and social wellbeing.

## Introduction

Oral Potentially Malignant Disorders (OPMDs) are those lesions and conditions that have an increased potential for malignant transformation and are risk indicators of future malignancies [1]. A wide range of conditions that predispose oral mucosa to malignant transformation are considered as OPMDs [2] [3]. The term OPMD can be holistically defined as ‘a group of disorders of varying aetiologies, usually tobacco; characterized by mutagen associated, spontaneous or hereditary alterations or mutations in the genetic material of oral epithelial cells with or without clinical and histomorphological alterations that may lead to oral squamous cell carcinoma transformation.’ [4].

The most important OPMDs that have malignant potential are Erythroplakia, Oral Leukoplakia (OL), Oral Lichen Planus (OLP), Oral Submucous Fibrosis (OSF) and Actinic Keratosis [1, 5]. Erythroplakia, OL, and OSF are habit-related OPMDs with tobacco users being at increased risk of developing Erythroplakia and OL while OSF is associated with areca/betel nut usage [6, 7]. On the other hand, OLP and actinic keratosis are non-habit related, being immunologically mediated and sun exposure related potentially malignant disorders respectively [8, 9]. The most prevalent forms among the above-discussed OPMDs are OL, OLP and OSF [10], which are associated with discomforting symptoms including pain, burning sensation and limited mouth opening [11]. The bodily pain and dysfunction caused by OPMD may have implications on emotional and social wellbeing. Further, the fear of malignant transformation of the OPMD can cause psychological distress [12]. Therefore, the impact of OPMD on an affected individual’s life is multi-dimensional, like that of oral or most other malignancies. Patients diagnosed and treated for oral malignancies have been found to experience poor quality of life (QoL), and many now urge that QoL assessments be a standard criterion for evaluation of oral cancer patients and their response to treatment [13, 14].

The literature on QoL assessment in OPMD is, however, scanty [12], which might be partly due to the non-availability of a QoL measure specific for OPMD. QoL assessments are mostly made by use of questionnaires [15], being part of a Patient Reported Outcome Measure (PROM). However, a PROM is not just a questionnaire to collect patient opinions but intends to measure specific concepts in a standardised manner [16]. A PROM demonstrates a patient’s perspective of function, well-being and QoL associated with their disease [17]. Understanding by health care providers of the effects of disease on the everyday life of their patients is frequently poor [18], warranting the use of PROMs.

Development of a PROM needs to abide by several considerations, one of which is that the content should be derived from interviews with relevant patients for the measure to be relevant and acceptable to the prospective patients [16]. This paper presents the findings from the qualitative analysis of patient interviews, being the first step towards our goal of developing an appropriate QoL questionnaire for people with OMPD.

## Material and methods

This study conforms to Consolidated Criteria for Reporting Qualitative research (COREQ) guidelines for reporting qualitative research [19]. All the patients with OPMD (OL, OLP, and OSF) undergoing treatment at the oral medicine clinics of Panineeya Institute of Dental Sciences & Research Centre, Hyderabad, India, during May 2014—July 2014, were approached face to face and were invited to participate. The diagnoses for OL, OLP, and OSF were made by qualified specialists in oral medicine based on clinical and histopathological examinations. All the eligible patients were provided with information sheet; all those who were approached provided written informed consent and participated in the study. A thorough case history was taken which also involved the collection of socio-demographic information. Disease severity was graded for OSF based on the degree of mouth opening, determined by Lai et al. [20]. The scoring system proposed by Escudier et al., [21] was used for grading OLP, which takes into account the extent of involvement, the severity of tissue damage and pain. The grading of OL was based on size and homogeneity of the lesion [22]. Patients with concurrent oral mucosal conditions such as recurrent ulceration, overt oral malignancies or with odontogenic infections were excluded. Ethics approval was obtained from the Human Research Ethics Committee of Griffith University (DOH/14/14/HREC) and the Ethics Committee of Panineeya Institute of Dental Sciences and Research Centre (Ref#00125).

**Fig 1:**
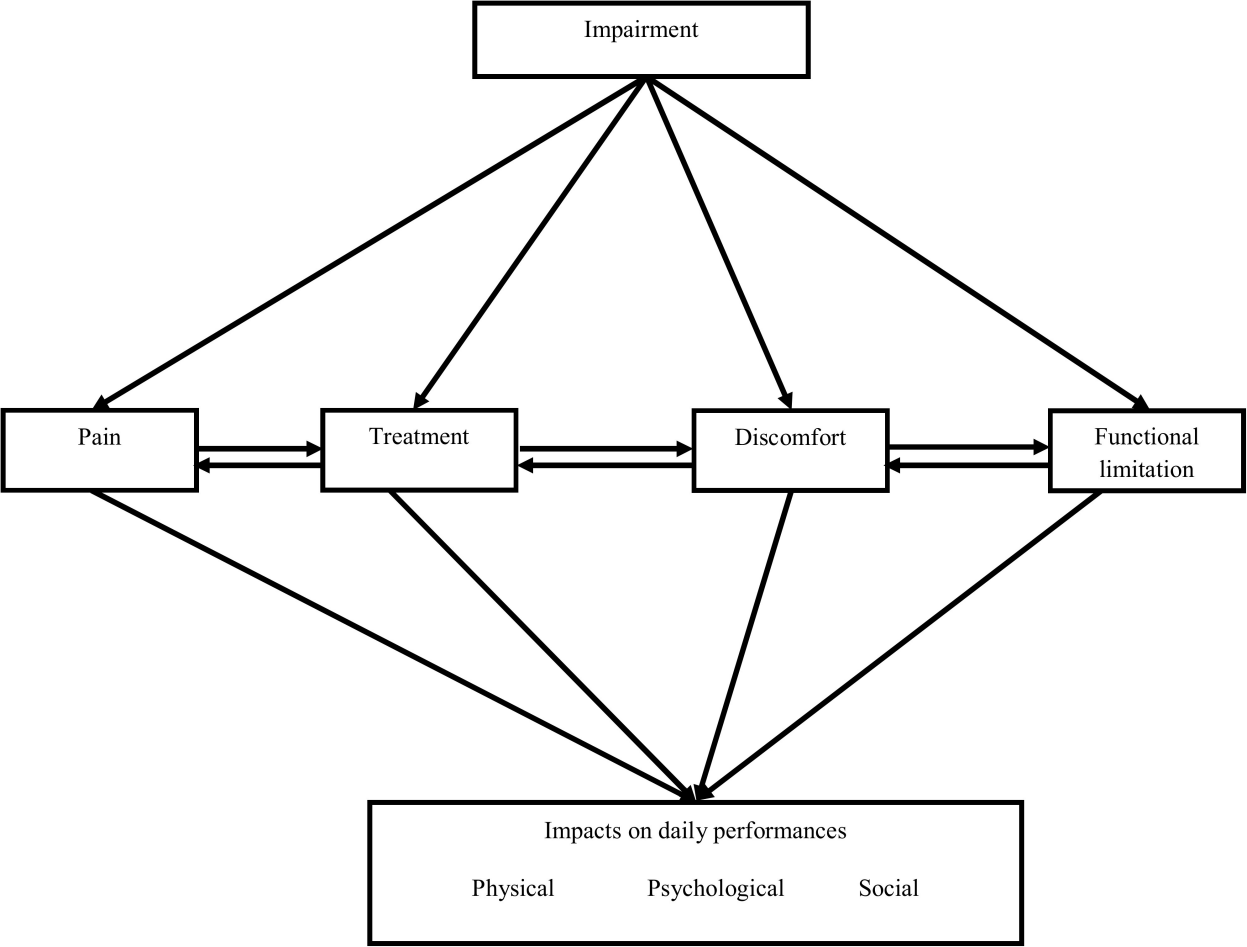
Conceptual model adapted from Oral Impacts on Daily Living theoretical framework of Adulyanon and Sheiham.

The theoretical framework (Fig 1) that formed the basis for the development of this instrument has been reconceptualized from the Oral Impacts on Daily Performances (OIDP) theoretical framework. The OIDP framework is based on the Locker’s model which has been modified for dentistry from World Health Organization's (WHO) International classification of impairments, disabilities, and handicaps. This model measures the ultimate impact of oral health on the ability to perform daily activities that include physical, psychological, and social performances [23]. Also, the effect of treatment on daily performances has been added to the model as there is no single standard treatment available for most of the OPMDs [24–26], and the treatment procedure is time-consuming, and the conditions might relapse which ultimately impacts the performances on daily living. A modified grounded theory approach was used incorporating clinical knowledge of experts along with the patient experience [27] in developing the QoL instrument for OPMD patients.

**Fig 2:**
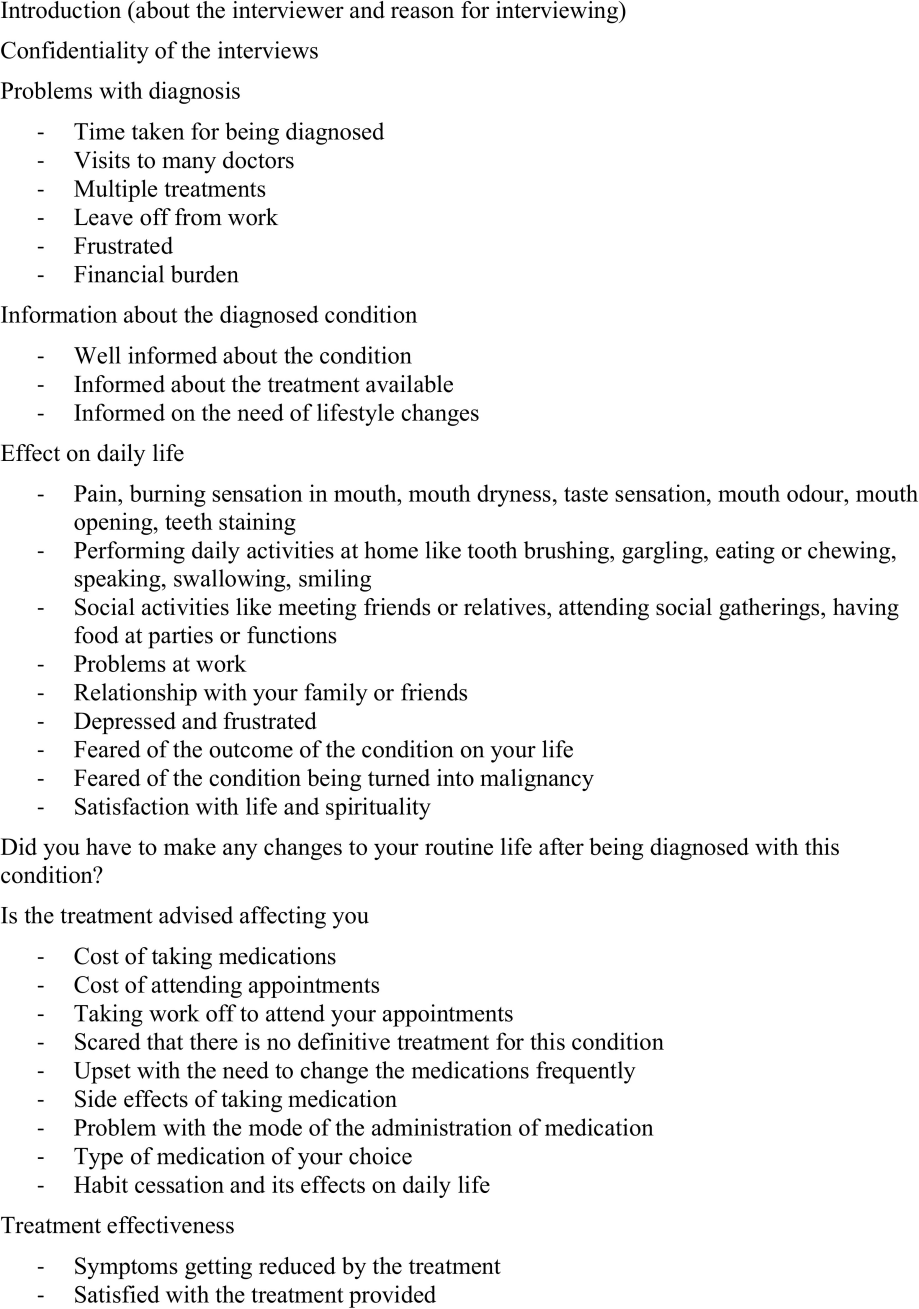
Interview guide used for conducting interviews and focus group discussion.

Consequently, an interview guide (Fig 2) was prepared after a thorough literature review that included the development of disease models, and with inputs from clinicians. This guide covered a wide range of topics that are related to OPMD patients. Pilot testing of the interview guide and mock interviewing were conducted with five patients to test if the interview guide is appropriate, which also served to rehearse the interviewer. Also, ten clinicians were approached to know about the aspects of daily life that are affected in OPMD patients using a Delphi technique.

An audit trail was maintained which comprised documentation of field notes along with the description of the study setting and population. In addition, data analysis, synthesis and methodological processes were recorded.

Qualitative data were obtained by conducting 18 one-to-one interviews with patients with a diverse range of characteristics that lasted for 20–40 minutes, and from three focus group discussions which took more than an hour each. Focus groups involve discussions in which a small group of informants, in this case, OPMD patients, are guided to talk freely and spontaneously about themes considered important to the investigation by a facilitator [28]. Group mix was kept to a minimum in the first two focus groups. The first and second groups comprised OLP and OSF patients respectively while the third group had patients with mixed conditions.

Personal interviews were conducted by the principal author (JT) while focus group discussions involved one moderator (JT) and one facilitator (SK). The interviewer was a female Ph.D. student who has received training in qualitative research and also an experienced oral physician. No attempt was made to establish a relationship with the patients before commencing the interviews. Interviews and focus group discussions were conducted in the Telugu language (the native language of the study population) in non-clinical settings. Non-participants (eg., relatives) were not allowed to remain while conducting the interviews or focus group discussions to ensure that patients felt comfortable. Interview sessions were recorded with a digital audio recorder while focus group discussions were both audio and video recorded. Handwritten notes were also taken during the discussions. As and when the recording was obtained, it was transcribed and translated using a parallel transcription method [29] whereby both transcription and translation are performed at the same time. This was performed by the chief investigator (JT) and was re-checked for accuracy several times. In addition, another investigator (SK) re-checked, cleaned and reviewed all the transcripts. During transcription, consideration was given to use the words and perspectives reported by the patients and few randomly chosen transcripts were shown to the patients for their comments. A saturation table (S1 Table) was prepared as and when the data from each patient were transcribed and this was monitored continuously to check for data adequacy. For achieving saturation, a combination of a saturation table and code book was used [30]. The transcripts were read, re-read and scrutinised diligently to identify re-occurring themes and subthemes. A random sample of transcripts was chosen and compared to the voice records to ensure quality and descriptive validity [27]. The English transcripts were analysed using NVivo software (QSR International’s NVivo 10) by two coders independently. A code book was prepared which defines each code along with its rules of application to enable reliable coding by two different coders [30]. To compare the number of times coding was done by the coders for each node, a tree map was created in Nvivo. Inter-coder reliability was calculated for each theme (node) and overall coding using percentage agreement and the Kappa statistic. Kappa values of 0.41–0.60 are considered moderate agreement, >0.60 is substantial agreement while values <0.41 are fair to poor agreement [30][31]. Both weighted and unweighted Kappa coefficients were calculated, weighted Kappa was calculated based on the size of the source from which the coding had been done.

## Results

A total of 32 OPMD patients participated in this study. Data saturation was achieved in the 11^th^ interview. Patient characteristics are presented in Table 1. The age of the study population ranged from 17–67 years, and 59% (n = 19) were males. More than half (62.5%) of the study population had less than 12 years of formal education and most of the patients (72%) belonged to semi-skilled/unskilled occupations or were unemployed.

**Table 1:** Characteristics of the patients.

Age	Gender	Condition	Severity of the condition
38	M	OSF	C
67	F	OLP	4
20	M	OSF	D
49	F	OLP	5
49	M	OL	Stage 1
36	M	OSF	D
41	F	OLP	5
20	M	OSF	C
22	M	OSF	C
25	M	OSF	D
36	F	OLP	3
55	F	OLP	5
17	F	OLP	10
44	M	OL	Stage 1
48	M	OL	Stage 2
35	M	OL	Stage 4
37	M	OL	Stage 2
38	F	OL	Stage 2
46	F	OLP	4
45	F	OLP	4
58	F	OLP	7
49	F	OLP	4
45	F	OLP	6
28	M	OSF	C
32	M	OSF	A
28	M	OSF	C
34	M	OSF	A
35	M	OSF	B
40	M	OLP	4
45	F	OLP	5
32	M	OL	Stage 1
37	M	OL	Stage 3

A–Mouth opening of >35 mm; B–mouth opening of 30–35 mm; C–mouth opening of 20–29 mm; D–mouth opening of less than 20 mm; L1C1 –Stage I; L2C1 –Stage II; L2C2 –Stage III.

OSF–Oral submaleucous fibrosis; OLP–Oral lichen planus; OL- Oral leukoplakia.

From the data analysis, four main themes were identified: (1) Difficulties with diagnosis and knowledge of the condition; (2) physical impairment and functional limitation; (3) psychological and social well-being, and (4) effects of treatment on daily life. Maximum coding occurred for the sub-theme “physical impairment” (91 coding references) followed by “functional limitations” (73 coding references). Table 2 compares the codings for each theme produced by the two independent coders. Kappa agreement for all the nodes (both weighted and unweighted) ranged from 0.53 (physical impairment and functional limitation) to 0.78 (psychological and social wellbeing). The overall Kappa agreement for all the themes together was substantial (0.66 –unweighted and 0.65 –weighted). The following paragraphs demonstrate the themes with selected direct quotes from the patients.

**Table 2:** Percentage agreement and Kappa coefficient between the coders for various nodes.

	Kappa	Agreement (%)
Average for node “Difficulties with diagnosis and knowledge of the condition (Unweighed)”	0.55	97.67
Average for node “Difficulties with diagnosis and knowledge of the condition (Weighed by source size)”	0.59	97.79
Average for node “Effect of treatment on daily life (Unweighed)”	0.65	98.54
Average for node “Effect of treatment on daily life (Weighed by source size)”	0.64	98.60
Average for node “Physical Impairment and Functional limitation (Unweighed)”	0.55	98.17
Average for node “Physical Impairment and Functional limitation (Weighed by source size)”	0.53	98.01
Average for node “psychological and social well-being (Unweighed)”	0.78	97.72
Average for node “psychological and social well-being (Weighed by source size)”	0.77	97.90
Average for all nodes & sources (Unweighted)	0.66	98.07
Average for all nodes & sources (Weighted by source size)	0.65	98.13

### Difficulties with diagnosis and knowledge about the condition

This theme comprised difficulties encountered by patients before being given a definitive diagnosis for their oral condition, plus concerns they had regarding the lack of clear information on the nature of their oral condition and treatment options. It comprised of some sub-themes: multiple referrals, the frustration of not being diagnosed and knowledge about the condition and its treatment.

A large proportion of participants (47%) reported that they had gone through multiple referrals and appointments, all in vain. For instance, one of the patients who was a chronic sufferer of OLP stated:

“That’s a very long list; I have spent approximately half of my life visiting doctors for this problem. I have visited many doctors; I do not even remember the number of doctors I have visited for this problem. There is no treatment which I have not taken; each doctor tried his choice of medication on me, but no treatment was effective” (58 years old female OLP patient).

The period before being given a firm diagnosis was stressful for some patients (41%), and was described as frustrating:

“That phase of 6 months was the worst in my life; I was frustrated and under stress, as no doctor could relieve my pain, the three doctors I have consulted suggested three different kinds of treatments and none could permanently cure my pain. I couldn’t think of anything other than my mouth pain both at home and office” (55 years old female OLP patient)

However, once diagnosed, most patients (63%) were happy with the knowledge they were given on their oral condition:

“On my first visit itself, the doctor here told me that this mouth problem is due to gutkha chewing. I also came to know if I continue with the habit it can turn into mouth cancer. I was explained about the treatment method, and doctor said that the treatment for my mouth condition is time taking” (22 years old male OSF patient)

### Physical impairment and functional limitations

This was the most important theme for many patients and most of the interview time was spent discussing this theme. Two sub-themes emerged, namely physical impairment and functional limitations.

In general, patients with OLP and OSF had several complaints with regards to physical impairment and functional limitations. Burning sensation on eating spicy food was the most distressing complaint, and this was reported by more than two-thirds of all patients with OSF and OLP:

“Do not ask about that (*effect of mouth condition on eating*). Sometimes I feel that I am eating food just for the sake of living, I do not enjoy eating my favourite foods anymore, and I find the taste of all foods similar. I have pain and also feel some burning sensation on eating food, the burning sensation in the mouth gets worse on eating spicy food. I also feel dryness in the mouth, and I do not appreciate the taste” (20 years old male OSF patient).

“I have anyway stopped eating my all-time favourite biryani (*an Indian dish*) since 2–3 years because I get the severe burning sensation when I eat spicy food. I have become selective these days while eating food, this (*pointing to his mouth*) has made my life difficult as we are always served spicy food everywhere we go…In my community, hot and spicy food is part of life” (38 years old male OSF patient).

Although patients did not report the direct pain in the lesion, few expressed pain and agony associated with the lesion. For instance, one patient with OSF described:

“More than the burning sensation and mouth opening, pain in my ear is very severe, and I cannot think nor do anything. I don’t even sleep these days; the medicines relieve pain only for half an hour” (25 years old male OSF patient).

Another patient explained:

“I get pain while brushing the teeth when the toothbrush touches the place where this problem is (*points to the lesion on the buccal mucosa*), I have even tried some mouthwash, the pain it causes is worse…The best method I have adopted is using a finger to clean my teeth” (17 years old female OLP patient).

Other than burning sensation, difficulty in opening the mouth was the second most common complaint of patients. All the OSF and few OLP patients reported issues with limited opening of the mouth. However, the reported consequence of limited mouth opening varied between the patients. For instance, one patient said:

“The only problem I have is in opening the mouth, I think my mouth doesn’t open full like others, see I can hardly put two fingers into my mouth (*inserts two fingers to show*). I face difficulty in eating big bites and foods like paani poori (*Indian snack*) because of not being able to open the mouth widely” (34 years old male OSF patient) explaining how his limited mouth opening is preventing him from having particular varieties of food.

Another patient described a peculiar anecdote that has caused embarrassment due to the limited mouth opening:

“I am unable to blow air into balloons because of the inability to open my mouth widely. Recently, I had to face an embarrassing situation because of this. I have been to my neighbours’ son’s birthday party, and they wanted me to help in blowing the balloons for decorating. Although I tried, it was very tough for me to blow air into a balloon and it was more humiliating when they started asking about the reasons for not being able to open the mouth normally” (36 years old male OSF patient).

### Psychological and social wellbeing

Broadly, two sub-themes (psychological wellbeing and social wellbeing) could be recognized under this theme. Most patients (81%) reported their mouth condition as having a debilitating effect on their psychological wellbeing, approximately half of the patients reported being frustrated and depressed:

“I feel frustrated, depressed and sometimes get very angry because of my mouth condition. If it was pain only, I could have taken it but see I had to change my whole life because of this problem” (45 years old female OLP patient).

Another patient who was an oral physician herself revealed:

“Though I used to see OLP patients and feel empathy for them, I never thought this condition would be so painful that it makes you feel frustrated until I have experienced this. Initially, I had gone through a rough phase of depression, just wondering, why me? I have now come over it but look how much important role it plays in your life; I had to make changes to what I eat at home and think twice before going to a restaurant as I am anyway not going to enjoy food” (36 years old female OLP patient).

During the interviews, we made sure not to mention cancer, but all patients were aware of the potential of their oral condition developing into a malignancy and revealed their fear of getting mouth cancer:

I was very much worried when I got to know that this white patch in my mouth could turn into cancer. I am just praying God that this mouth problem should be cured as soon as possible (32 years old male OL patient).

For some (9%), being diagnosed with a potentially malignant condition was a revelation:

“I was initially very depressed and worried when my doctor told that I may get mouth cancer. I used to think that I am an educated fool who is addicted to gutkha in spite of knowing about its harmful effects on health. I thought if I do not give up gutkha then I would definitely get mouth cancer and would have wasted my life and career” (20 years old male OSF patient).

There was not only psychological despair: many patients (59%) had compromised their social interactions. For example, one patient stated:

“I do not like eating and attending parties as it is not easy for me to eat normally like others because of my mouth opening and I don’t like to get embarrassed” (20 years old male OSF patient).

### Effect of treatment on daily life

There were four sub-themes under this theme: financial impact of treatments; difficulty in keeping appointments; satisfaction with treatment and impact of habit cessation. The sub-themes of the financial impact of treatments and difficulty in keeping appointments were interrelated. The treatment of oral potentially malignant disorders is usually of long duration and involves multiple appointments so that keeping up with the appointments was an issue for patients. Many of these patients were of low socioeconomic status so that attending an appointment incurred loss of salary and induced financial stress:

“I am worried about coming to the hospital every week. My family runs with my work. For each visit, I will have to leave my work and travel for 2 hours” (49 years old male, OL patient).

Although there were contrasting reports on satisfaction with the treatment provided, the matter was discussed by most patients. One patient was very satisfied which can be understood from her words:

“After an almost eight years of agony, I have started feeling better now. I do not know how to thank my doctor; my life would have gotten better by now if I had taken this treatment before” (58 years old female OLP patient).

In contrast, some were dissatisfied with the outcome of the treatment and the mode of treatment:

“I do not understand what is happening with me. I have never missed an appointment, but I see no improvement. On top of this, the injections are so painful and unbearable that I get apprehensive to come for my appointment” (32 years old male OSF patient).

Lastly, some of the OSF and OL patients expressed considerable difficulties in coping with the withdrawal of chewing habits:

“I was addicted to gutkha for almost ten years, so it gets difficult to control crave. I sometimes feel very depressed, tense and frustrated because of this, so now I take chewing gum to compensate for this. Sometimes, I also get a headache and feel unwell, but I am hoping to overcome these problems soon” (20 years old male OSF patient).

## Discussion

In this study, semi-structured interviews were conducted with patients diagnosed with common OPMD; OL, OSF, and OLP. Although, we intended to include patients with Erythroplakia, no classic cases presented. In this research, we initially conducted a systematic review [12] and observed a paucity of literature on QoL issues in OPMD patients: there is no condition-specific Patient Reported Outcome Measures (PROM) currently available for OPMD patients. Chronic Oral Mucosal Disease Questionnaire (COMDQ) developed by Riordain et al., [32] in Ireland was found to be valid and reliable [33] for evaluating the QoL in patients with various oral mucosal lesions. However, it might not be relevant to patients with OPMDs other than OLP as cases of OL and OSF were not evaluated. [32].

PROMs have gained considerable importance recently. Their role in clinical practice is increasingly recognised as it reveals the patient’s perspective on the impact of illness on his/her functional status and overall well-being [34]. Further, PROMs are particularly important for patients with chronic disease, where survival may not be the most relevant outcome of therapeutic management [30]. This study involves our first step towards the development of an OPMD QoL questionnaire, particularly for the three common and important OPMDs; OL, OSF and OLP.

We have used both interviews and focus group discussions to collect data in this study. Both have their share of advantages and disadvantages. Personal interviews help in comprehensively attaining information on an individual’s personal experience, but it doesn’t offer peer comparison. On the other hand, focus groups facilitate patients to share their experiences by using others ideas as cues but sometimes one strong group member can influence the views of the entire group [27]. Semi-structured interviews were preferred over structured and unstructured. Semi-structured interviews are common formats in health care research and practice as they offer flexibility when compared to structured interviews and are less time consuming than unstructured interviews. Moreover, through semi-structured interviews, it is possible to uncover information important to patients which might have been previously thought insignificant by investigators [35].

Sample size in this study was adequate as saturation was achieved with the 11th interview itself. In qualitative studies, the sample size is dependent on data adequacy. Further, interview quality and patient diversity are more important than the absolute number of the participants [30]. Patients with a diverse range of characteristics, i.e., different types of OPMDs, levels of disease severity, age and education level were considered.

A segmentation analysis [36] for homogeneity of the focus groups was performed, the composition of the first two groups was kept homogenous as this increased the likelihood of interaction between the participants which is key to successful focus discussions [37]. On the other side, the composition of the third group was deliberately kept mixed in relation to gender, socioeconomic status, and disease diagnosis. This is because focus group discussions can sometimes become unproductive when all participants have similar perspectives [36].

Three (physical impairment and functional limitation; psychological and social wellbeing, and effects of treatment on daily life) of the four themes identified are similar to those identified by Riordain et al in the development of the COMDQ [32], namely biopsychosocial issues, treatment limitations and side effects.

The pre-diagnosis phase was very traumatic for most patients as they had to pay many visits and undergo different kinds of treatment, often with no relief. This is because of a general lack of knowledge concerning OPMDs and other oral mucosal lesions among many dentists and medical professionals [8]. A study in one district of India found that more than 70% of traditional medicine practitioners in India were unaware of the clinical appearance of early cancer and OPMD [38].

Burning sensation while having food was the most stressful problem reported by many patients; although some reported pain only while performing oral hygiene procedures. In addition, limited mouth opening was also widely reported. Both burning sensation and mouth opening could demand an alteration in an individual’s eating habits which could influence his/her emotional well-being and social activities as reported by many of our patients. Psychological distress in patients with chronic conditions is well documented and can have a greater impact on QoL than physical and functional limitations [39]. Riordain et al., [32] in their qualitative analysis also found pain and discomfort as the foremost problems in oral mucosal disease patients.

Fear associated with the possibility of their condition transforming into mouth cancer was prevalent in our participants. The term cancer is itself associated with fear and stigma in India [40], and cancer is perceived as fatal by most people irrespective of its specific prognosis [40, 41]. Many of our patients reported frustration, attributable to the chronic nature of the conditions, with delayed diagnosis and no specific effective treatment. Two relatively recent reviews have concluded that there is no reliable evidence on the effectiveness of various treatment modalities on the management of OSF [26, 42]. Also, for OLP, though corticosteroids have been the mainstay of treatment for decades, and a range of alternative treatments have been tried, a recent Cochrane review has found no specific treatment of OLP to be more consistently effective or even better than no treatment [25]. Similarly, although habit intervention and good nutrition are essential, there is no proven way to prevent malignant transformation of OPMD: adverse effects and relapses are common [43].

‘Physical impairment and functional limitations’ was the most important theme for many of the patients. However, the impacts of OPMD also extended beyond physical impairment and functional limitations to aspects of daily living, notably psychological and social wellbeing. These findings emphasise the need to consider patient perspectives when making clinical decisions rather than relying solely on the clinician’s judgement based on physical signs and symptoms.

The themes that emerged were related to the framework that was conceptualized. The data obtained from the interviews and focus group discussions in this qualitative study were used to generate the items in the questionnaire which were reduced and later scaled to develop an OPMD QoL instrument [44]. We believe this instrument can be used in patient populations in other countries after undergoing cross-cultural adaptation.

## Supporting information

S1 Table Data saturation table describing the emergence of new codes with the interviews. (DOCX)
